# The Effects of Radix Astragali Water Abstract on Energy Metabolism in Rat Yang-Deficiency Cold Syndrome Model through PPAR Signaling Pathway

**DOI:** 10.1155/2018/9194362

**Published:** 2018-11-01

**Authors:** Wanchen Yu, Haijun Zhao, Xin Zong, Xuming Ji, Xiaochun Han, Yanfang Wang, Yanan Zhang, Ke Ma, Ning Cui, Shijun Wang

**Affiliations:** College of Traditional Chinese Medicine, Shandong University of Traditional Chinese Medicine, Jinan 250355, China

## Abstract

Radix Astragali (RA) herb with warm property and significant “tonifying qi” effects is indicated for the syndrome of internal cold due to Yang deficiency. The purpose of this research was to explore effects of Radix Astragali (RA) through PPAR signaling pathway on gene expression profiles related to energy metabolism in rats with the Yang-deficiency cold (YDC) syndrome, for identifying the pathological mechanism of Yang-deficiency cold (YDC) syndrome and the effects mechanism of RA. The results indicated that RA could significantly increase body weight (BM), cold and heat tendency (CT), overall temperature (OT), rectum temperature (RT), toe temperature (TT), energy intake (EI), and V(O_2_)/V(CO_2_) ratio (which indicates basal metabolism, BM) (P<0.05), enhancing the depressed metabolic function in YDC syndrome model rat. Our data also indicated differentially expressed genes (DEGs) related to energy metabolism involving lipids, carbohydrates, and amino acids metabolic process; the expression of CPT-1 and FABP4 (ap2) was improved; PPAR, Glycolysis, Wnt, cAMP, MAPK, AMPK, and fatty acid degradation signaling pathway may be related to energy metabolism. However, the Chinese herbal medicine RA plays a certain role in promoting the metabolism of substances and energy in rats by its warming and beneficial effect. Our results suggest that the mechanism underlying the function of RA may take effect through the regulation of PPAR signaling pathway and related gene expression. Lipids, carbohydrates, and amino acids metabolic process may be affected to adjust the reduced metabolic function in the model animals. In general, results indicate that RA could promote energy metabolism in rats with the YDC syndrome via PPAR signaling pathway regulating the expression of CPT-1 and FABP4 (ap2), which reflected the warm and qi tonifying properties of RA.

## 1. Introduction

Yang-deficiency cold syndrome (YDCS) has long been a common and frequently occurring clinical disease. In the modern period, YDCS is even more commonly seen in multiple clinical disorders. Most acute diseases (such as craniocerebral injury combined with lung infection, hepatic encephalopathy, etc.) show this syndrome. YDCS also frequently appears in gynecological and digestive diseases and even more often in chronic diseases (such as type II diabetes) and advanced cancer [1–3]. Currently, there are many studies focusing on YDCS. However, the pathological mechanism is still not clear [4, 5]. “973” project is the most important fund of Chinese government in basic theory research. Our research group luckily applied for this project “Traditional Chinese Medicine Drug Properties Theory Related Basis Problem Study” (2007CB512601), the chills and fever of Chinese traditional medicine in the process of pharmacodynamics research. Under the guidance of traditional Chinese medicine theory, we established a mature animal model that mimics the characteristics and symptoms of YDCS. According to the previous results, the test on gene expression profile of the liver demonstrated that the cytokine network dysregulation is represented by carbohydrate and lipid metabolism imbalance in YDCS model rats [6, 7]. According to the preliminary experimental results of our research group combined with literature research and clinical observation, it is speculated that the occurrence of YDCS may be closely related to the abnormal changes of energy metabolism. PPAR is one of the main pathways for energy metabolism and plays a crucial role in changing energy metabolism. Therefore, this experiment will use both macroscopic observation and microscopic detection to verify the mechanism of YDCS and the role of PPAR signaling pathway along with its target genes CPT-1 and FABP4.

Promotion or inhibition of carbohydrate and lipid metabolism is mainly affected by the target genes CPT-1 and FABP4 in the PPAR signaling pathway. Peroxisome proliferator-activated receptor (PPAR) is a subfamily of nuclear hormone receptors; three isoforms of PPARs have been identified, namely, PPAR-*α*, PPAR-*β*/*θ*, and PPAR-*γ*. PPAR-*α* and PPAR-*γ* are mainly involved in regulating glucose and lipid metabolism, insulin sensitivity, and immune response. Therefore, specific focus on the therapeutic potential of PPAR modulators in gene regulation and metabolic diseases may become a promising direction of developing treatment for metabolic syndrome [8–11]

CPT1 (carnitine palmitoyltransferase 1B) is an integral membrane protein [12]. It is the first element and rate-limiting step of the carnitine palmitoyltransferase system and also an essential step in the beta-oxidation of long-chain fatty acids [13]. In addition, the important role of CPT1 in fatty acid metabolism makes it a potentially useful enzyme in developing the treatment for many other metabolic disorders [14]. CPT1 is inhibited by malonyl-CoA. This inhibition is an ideal target to regulate CPT1 for the treatment of metabolic disorders [15]. FABP4 (adipocyte-fatty acid-binding protein 4, also known as aP2) is involved in the regulation of glucose and lipid metabolism. FABP4 is also capable of resisting the development of atherosclerosis and improving the sensitivity of insulin [16]. Along with many other previous publications, people showed that FABP4 has a close relationship with metabolic syndrome, inflammation, atherosclerosis, obesity, insulin resistance, diabetes mellitus, hypertension, cardiac dysfunction, cardiovascular events, etc. [17]. These two target genes, namely, CPT-1 and FABP4, are in close relation to the activation of PPAR signaling pathway.

This important multiple syndrome is often ignored in the early stages of many diseases [1–5], and the efficacy of treatment is poor. Therefore, a highly effective and mild Chinese medicine that can better suit early treatment and can be taken in long-term is urgently needed to be applied in the treatment of YDCS. It is helpful to suppress the development of diseases and improve the prognosis of diseases. Our research group studied the medicinal properties of a mild beneficial traditional Chinese herbal medicine, RA membranaceus. RA is a commonly used TCM that has sweet taste with warm property. It belongs to lung and spleen meridians and it has such functions as warming the interior and dispelling cold, strengthening and uplifting Yang, and warming and activating meridian; because of its ability to replenish Qi and Yang, RA is often used clinically to treat YDCS caused by insufficient Yang. Modern pharmacology studies show that RA can enhance energy metabolism and immunity. The mechanism of its action may be through the PPAR signal pathway, which regulates energy metabolism. Therefore, our investigation took the change in PPAR signal pathway affecting energy metabolism as a starting point.

We conducted and explained our study mainly through the following aspects: observation of the overall pharmacodynamics focused on energy metabolism; detection and real-time monitoring of rat whole body surface temperature using forward looking infrared (FLIR) cameras; monitoring of rat energy metabolism changes and trends in a 24-hour time frame; and analysis of the possible mechanism of warmth production using RNA-seq transcriptome sequencing from the perspective of gene expression profiles.

## 2. Materials and Methods

### 2.1. Animals

Thirty-six specific-pathogen-free (SPF) grade, six-week-old Wistar rats (half male and half female), weighing 200±20 g, were acquired from Beijing Vital River Laboratory Animal Technology Co., Ltd. (Beijing, China), experimental animal license number: SCXK (Beijing) 2016-000140. All experiments were done in accordance with the guideline and regulation by the Administration Office of Laboratory Animals at Jinan, China. All experimental protocols were approved by medical Ethics Committee of Shandong University of Traditional Chinese Medicine.

### 2.2. Animal Model

Rats were acclimated for three days in our animal facility and then randomly divided into three groups: normal control group (NCG), YDCS model group, and RAWA treatment group (12 animals in each group). Model rats and RAWA treatment group were fed with TCM mixture every afternoon for two weeks to establish YDCS model.

The animal model were established using TCM mixture (Gypsum, Radix Gentianae, Cortex Phellodendri, and Rhizoma Anemarrhenae mixed in a 2:1.2:1:1.5 ratio [7]) according to the relevant literature [18, 19]. The mixture was enriched to 4 g/ml of water abstract and stored in 4°C for later use. The abstract was administered by gavage with the dose of 64g·kg^−1^·d^−1^, 4g/ml, 16ml/kg (the model group and the treatment group). The administration was performed once a day, fourteen consecutive days. The evaluation standards for the establishment of the YDCS model were based on previous experimental studies and with reference to the literature [20]. The partial least-regression method (PLS) was used to select indicators that can determine the degree of the deficient cold model: fecal texture, hair cleanliness, water intake (in positive correlation with the model), urine color, body weight, average toe temperature, and autonomic activity (in negative correlation with the model). Therefore, the standards for successful replication of the YDCS model are as follows: Rats have loose stools. Hair is dirty and messy. Water intake is large (macroscopic representation). There is weight loss. Basal (respiratory) metabolism (RER) is reduced without interference for 24 hours (changes in metabolism index). The anal temperature, toe temperature, and overall body temperature showed a decreasing trend (temperature decreased, and metabolism was significantly reduced). These are accurately consistent with the symptoms of the deficiency cold syndrome and its evaluation indicators have reached the standard. The deficiency cold model was successfully replicated in this experiment.

The BCG was given equal amounts of normal saline at the same time.

### 2.3. Preparation of RAWA

RAWA was prepared using RA membranaceus. The herb was cultivated in Gansu Province, China, and identified by Prof. Feng Li from the Department of Traditional Chinese Medicine, Shandong University of Traditional Chinese Medicine. RA membranaceus was boiled into water decoction. The concentration of the crude drug was 0.54g/ml and stored at 4°C.

### 2.4. Experimental Protocol

After the model was successfully established, RAWA was administrated for treatment, according to* People's Republic of China Pharmacopoeia* (2015 edition), and RA was administered at twice the dose used in adults (60g/70kg). Based on the equivalent dose coefficient algorithm, the rat equivalent dose was 6.3 times that of adults per body weight unit. The abstract was administered by gavage with the dose of 5.40g·kg^−1^·d^−1^, 0.54g/ml, 10ml/kg [21]. The administration was performed once a day, seven consecutive days [6]. The normal control group and the model group were given equal amounts of normal saline.

After the last administration, the animals were euthanasia. The livers were rapidly separated at 4° and frozen in liquid nitrogen.

### 2.5. Temperature Change Detection

7 days, 10 days, and 14 days after drug administration, the overall temperature (OT), rectum temperature (RT), and toe temperature (TT) of the experimental animals were examined using forward looking infrared (FLIR) cameras and DT-880B hand-held infrared body surface temperature rapid screening instrument. Each group of animals was tested alternately in parallel experiments.

### 2.6. Metabolic Rate Monitoring

Basal metabolism changes were automiaticly detected in rats for 24 hours by using LabMaster. Noninterfering basal metabolism monitoring was performed after the establishment of animal model following the administration of RAWA. Oxygen consumption, carbon dioxide production, respiratory exchange rate, and energy consumption were calculated by indirect calorimetry. The frequency and time of drinking and feeding were captured by high-precision sensors. The total activity of rats in cages was measured by beam sensors. Therefore, noninterfering basal metabolism changes and their significance in YDCS model group and RA treatment group can be analyzed.

### 2.7. Statistical Analysis

Data analysis was performed using SPSS 24.0 (IBM, LSD, P <0.05) and presented by mean ± standard deviation. F-test was used for the comparison of consecutive variables between groups. Chi-square test was used in the categorical variable group. Single-factor variation analysis of variance was used for comparison. And LSD test was used for further comparisons between pairs. Significant p-values were as follows: *∗∗* P < 0.01,*∗* P < 0.05 versus normal control; ^##^P <0.01, ^#^P <0.05 versus model.

### 2.8. Transcriptomic Sequencing and Analysis

Sample total RNA was extracted using TRIzol reagent (Ambion-1561), and DNA was digested using DNase. RNA integrity was assessed using the mirVana miRNA Isolation Kit (Ambion) of the Agilent 2100 Bioanalyzer (Agilent Technologies, Santa Clara, CA, USA). Briefly speaking, a sequencing library was generated using the TruSeq chain mRNA LT Sample Prep Kit (Illumina, San Diego, CA, USA) according to the manufacturer's instructions. Oligo (dT) magnetic beads are used to enrich eukaryotic mRNAs. Interrupting reagents are added to break the mRNA into short fragments. The interrupted mRNA is used as a template to synthesize one-strand cDNA using six-base random primers. A two-stranded synthesis reaction system is then used to synthesize the double-stranded cDNA, after which PCR is performed to enrich the cDNA template. Purify the PCR product (AMPure XP system). Using TruSeq chain type LT Sample Prep Kit and cBot cluster generation system to cluster exponentially encoded samples (Illumina). The sequencing library was then sequenced (Illumina HiSeq X 10) on an Illumina sequencing platform by collecting 150 bp of double-ended data. The entire experiment was conducted at Shanghai Quanmai Biotechnology Co., Ltd. (Shanghai, China). Raw reads are sequences in fastq format. The NGS QC Toolkit software [22] was used for quality control and removal of the linker. Based on this, low-quality base and N base reads were filtered out, resulting in high-quality clean reads. Clean reads were then aligned to the reference genome and reference genes of this species using Bowtie2 [23] or TopHat [24]] (http://tophat.cbcb.umd.edu/). The status of the sample was assessed by the genome and the rate of gene alignment. The result of the comparison is stored as a binary file, namely, a bam file. Using HTSeq [25] software to obtain the number of reads aligned with the gene in each sample. Cufflinks [26] software was used to calculate the gene expression quantity FPKM (Fragments Per kb Per Million Reads) [27], which is the number of fragments from each kilobase of a gene per million fragments. Then the clustering method is used to calculate the distance between samples, examine the similarity between the samples, and calculate the direct correlation of the samples. Generally speaking, the same type of sample can appear in the same cluster through clustering. Genes in the same cluster may have similar biological functions.

### 2.9. Functional and Pathway Enrichment Analysis of DEGs

Differential expression analysis was performed to identify differentially expressed genes among different control groups, YDCS model group, and RA treatment group (using the estimateSizeFactors function of DESeq (2012) R package [28] to normalize the data and using the nbinomTest function to compute the P-value and fold change values for the difference comparison; P-value<0.05 and fold change>2 or fold change<0.5 are set as the threshold of significant differential expression). Using the negative binomial distribution test to calculate the gene differential expression level DESeq software [29]. (http://bioconductor.org/packages/release/bioc/html/DESeq.html). Using NB (negative binomial distribution test) to test the significance of reads. The base mean value was used to estimate the expression level of the gene expression. GO function significance and KEGG pathway significance analysis were used to determine the major biological function or pathway affected by differential genes. Subsequently, alternative splicing analysis and SNP analysis (Single Nucleotide Polymorphisms) were performed on differential genes. By alternative splicing of one gene, multiple proteins may be produced. This can greatly increase protein diversity. Based on the sequencing data, we used ASprofile software [30] to detect the presence of alternative splicing in the sample. Further comparisons of the differential genes were performed by comparing the assembled gene with the gene annotation information of the reference sequence. This may reveal that the assembled gene will extend the 5′ or 3′ end of the gene annotation, thereby optimizing the gene structure. The new annotation information is compared with known annotation information to obtain new transcript information. With the above comparison results, new transcript predictions can be performed simultaneously. Assembly results must satisfy the following conditions to be considered as new transcripts: 200 bp or more from the existing annotation gene and no less than 180 bp in length. Based on the TopHat-Cufflinks analysis platform, reads were assembled using Cufflinks software and new transcripts were assembled and compared with the gene annotation information of the reference sequence by Cuffcompare software. Read data from raw-sequence RNA-seq are unloaded to ftp://ftp.ensembl.org/pub/release-84/fasta/rattus_norvegicus/cdna/Rattus_norvegicus.Rnor_6.0.cdna.all.fa.gz.

### 2.10. Quantitative Real-Time PCR Analysis

Quantification was performed with a two-step reaction process: reverse transcription and PCR. Each RT reaction has two steps.

The first step is 0.5 *μ*g RNA, 2 *μ*l of 4×gDNA wiper Mix, adding Nuclease-free H2O to 8 *μ*l. Reactions were performed in a GeneAmp® PCR System 9700 (Applied Biosystems, USA) for 2 min at 42°C. The second step is add 2*μ*l of 5 × HiScript II Q RT SuperMix IIa. Reactions were performed in a GeneAmp® PCR System 9700 (Applied Biosystems, USA) for 10 min at 25°C; 30 min at 50°C; 5 min at 85°C. The 10 *μ*l RT reaction mix was then diluted × 10 in nuclease-free water and held at -20°C. Real-time PCR was performed using LightCycler® 480 II Real-time PCR Instrument (Roche, Swiss) with 10 *μ*l PCR reaction mixture that included 1 *μ*l of cDNA, 5 *μ*l of 2× QuantiFast® SYBR® Green PCR Master Mix (Qiagen, Germany), 0.2 *μ*l of forward primer, 0.2 *μ*l of reverse primer, and 3.6 *μ*l of nuclease-free water. Reactions were incubated in a 384-well optical plate (Roche, Swiss) at 95°C for 5 min, followed by 40 cycles of 95°C for 10 s, 60°C for 30 s. Each sample was run in triplicate for analysis. At the end of the PCR cycles, melting curve analysis was performed to validate the specific generation of the expected PCR product. The primer sequences were designed in the laboratory and synthesized by Generay Biotech (Generay, PRC) based on the mRNA sequences obtained from the NCBI database. The expression levels of mRNAs were normalized to input the reference gene, e.g., GAPDH, ACTB, and were calculated using the 2-ΔΔCt method (Livak and Schmittgen, 2001).

## 3. Results

### 3.1. Model Evaluation

Based on preliminary experiments and literature studies, the standards for successful replication of the YDCS model are compared with the normal control group; the model rats mainly changed in the macroscopic symptoms, metabolic indicators, and temperature indicators.

**Figure 1:**
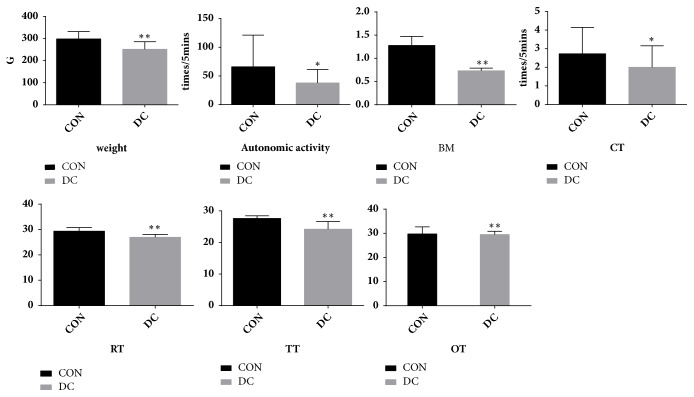
After the establishment of the YDCS model, compared with the blank control group, the changes shown in weight, autonomic activity, BM, CT, RT, TT, and OT in the model rats further explain the state and extent of YDCS. *∗*P < 0.05, *∗∗*P < 0.01 versus control group.

After the model was established, compared with the normal group, the YDCS model rats showed macroscopic symptoms: the stool was loose, the hair was dirty and messy, the amount of water intake was high, the spirit was wilting, and there were symptoms of deficiency cold syndrome. On the metabolic indicators, the body weight and basal (respiration) metabolism (RER) was significantly lower (P<0.01). The autonomic activity and CT were significantly lower (P<0.05). On the temperature index, RT, TT, and OT were significantly decreased (P<0.01) ( Figure 1).

### 3.2. Test Results on Metabolism-Related Macroscopic Indicators

In this study, macroscopic observations were made on the energy metabolism of YDCS model and RAWA treatment group rats in terms of symptoms, weights, automatic monitoring of basal metabolic changes in noninterfering environments, cold and heat tendency, temperature changes, etc.

### 3.3. Measurement of the Symptoms

The results are shown as follows: there were no differences between the initial status of each group. Compared with the normal control group, rats in the model group appeared to have an addiction of lying, a decrease in food and water intake, loose stools, pale and partial darkness on lips and paws, temperature drop, messy and dry hair, clear urine in large amount, etc. As shown in Supplementary Figure 1, compared with clinical patients, the YDCS stage was characterized by symptoms including clear urine in large amount, loose stools, pale face, pale tongue, white coating on the tongue, weak and delayed pulse, and preference to warmth and soft touching, etc. These symptoms are consistent with YDCS model rats. After 7 days of treatment with RA water abstract, the overall condition of the animals improved significantly. The total activity, food intake, and water intake increased. Weight of the YDCS model animals increased stools became moderate. Lips and nails turned white and pink. The overall, rectal, and toe temperature all rise. Hair became smoother and shiner. There was improvement in urine color, etc. (Supplementary Figure 2). The differences between normal control group, YDCS model group, and RAWA treatment group in hair and paw color of the rats indicate the improvement in the condition of YDCS model group rats.

### 3.4. Measurement of the Temperature

**Figure 2:**
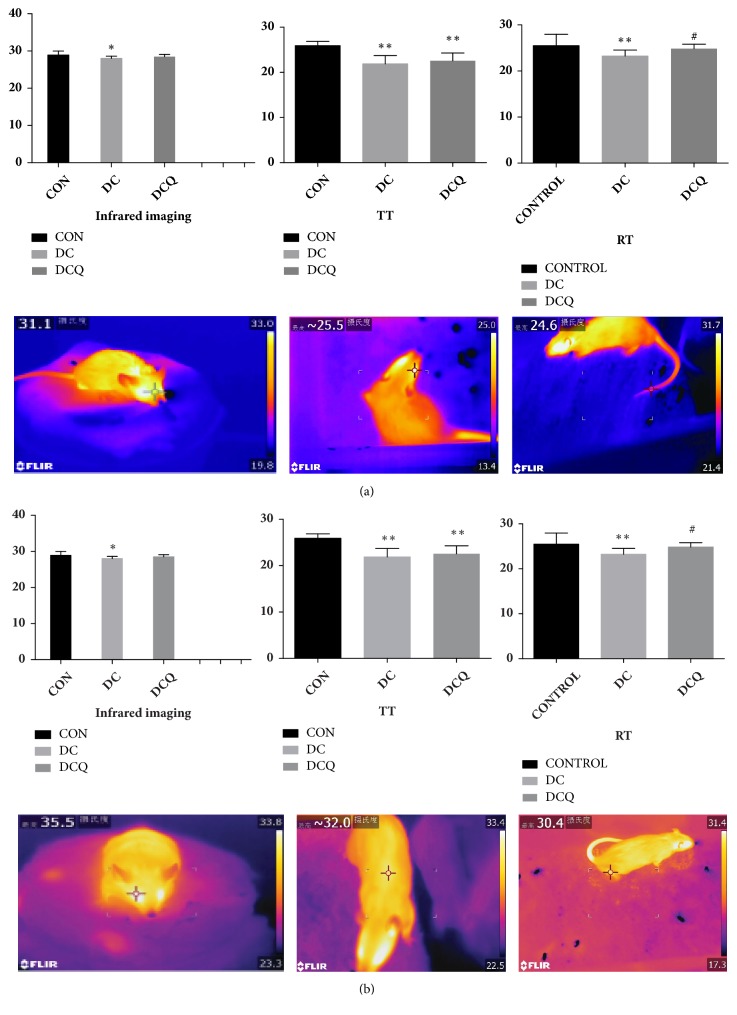
(a) The infrared imaging images of the rats in the YDCS model group and measurement of the temperature (OT, TT, RT). Data are represented as mean ±SD from three experiments. *∗*P < 0.05, *∗∗*P < 0.01 versus control group. #P < 0.05, ##P < 0.01 versus model group. (b) The infrared imaging images of the rats in the AWA treatment group and measurement of the temperature (OT, TT, RT). Data are represented as mean ±SD from three experiments. *∗*P < 0.05, *∗∗*P < 0.01 versus control group. #P < 0.05, ##P < 0.01 versus model group.).

According to the test of OT, RT, and TT, there was no difference between the initial status of three groups. Compared with the normal control group, the OT of the YDCS model rats was decreased (P<0.05). The average TT and the RT were significantly lower (P<0.01) as shown in Figure 2(a). However, in the rats treated with RAWA for 7 days, the OT and TT increased (P>0.05) and the RT increased significantly (P<0.05) as shown in Figure 2(b).

### 3.5. Measurement of the Body Weight, Cold and Heat Tendency, and Basal Metabolism

**Figure 3:**
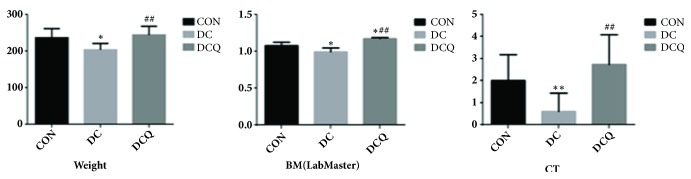
Measurement of the body weight, cold and heat tendency, and basal metabolism in YDCS model group. The results present averages of three independent experiments (mean ± SD). *∗*P < 0.05, *∗∗*P < 0.01 versus control group. #P < 0.05, ##P < 0.01 versus model group.

Effects on body weight, cold and heat tendency, and basal metabolism of rats were as follows: There was no difference in performance between the initial status of different groups. Compared with the normal control group, the body weight was significantly lower (P<0.05), the tendency of cold and heat was significantly colder (P<0.01), and the basal metabolism also dropped significantly (P<0.05) in the YDCS model group, as shown in Figure 3.

After seven days of treatment with RAWA, the body weight and basal metabolism of the rats were significantly higher than those of YDCS model group (tending towards neutrality and the cold-tolerant capacity increased), which was statistically significant (P<0.01). This shows that RA can treat YDCS efficiently and improve the reduced metabolism, which may be the intrinsic mechanism of its positive role in compensating Qi and Yang, as shown in Figure 3.

### 3.6. A Systematic Study of Molecular Mechanisms of YDCS and RAWA Effect Focusing on Energy Metabolism Using RNA-Seq Transcriptome Sequencing GO Functional and KEGG Enrichment Analysis of DEGs

**Figure 4:**
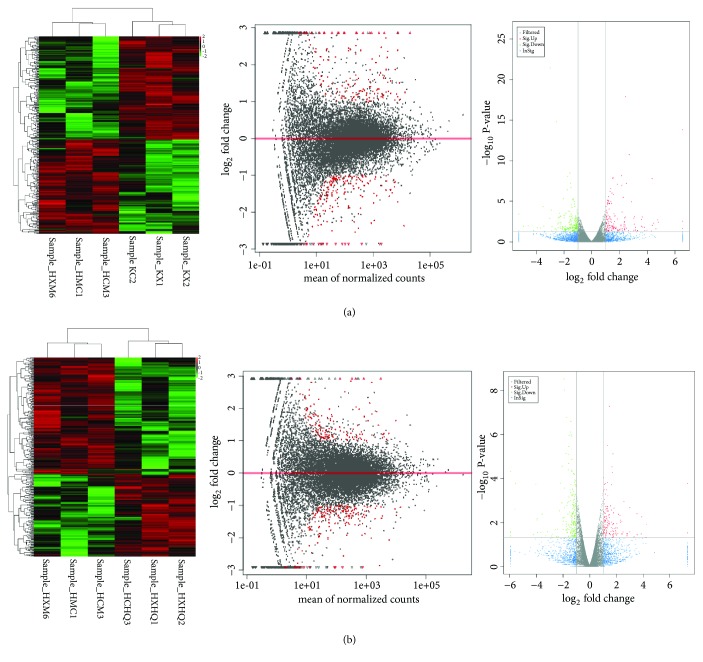
(a) Hierarchical clustering of the quantitative information from significantly changed gene expression by RNA-seq. Compared with the normal control group, the rat metabolism was related to YDCS; red indicates the high expression gene, and green indicates the low expression gene. (b) Hierarchical clustering of the quantitative information from significantly changed gene expression by RNA-seq, compared with YDCS group. The rat metabolism was related to the RAWA group; red indicates the high expression gene, and green indicates the low expression gene. The difference that generated is reflected in the MA diagram. The X-axis is the mean value of all samples used for comparison after standardization, and the Y-axis is log_2_ fold change. Red marked the difference genes in significance (according to differential screening conditions). The differences generated by comparison were reflected in the volcanic map, the gray and blue genes were nonsignificant differences, and the red and green genes were the significant difference genes. The horizontal axis is the display of log_2_ fold change, and the vertical axis is the display of log_10_ P-value.).

**Figure 5:**
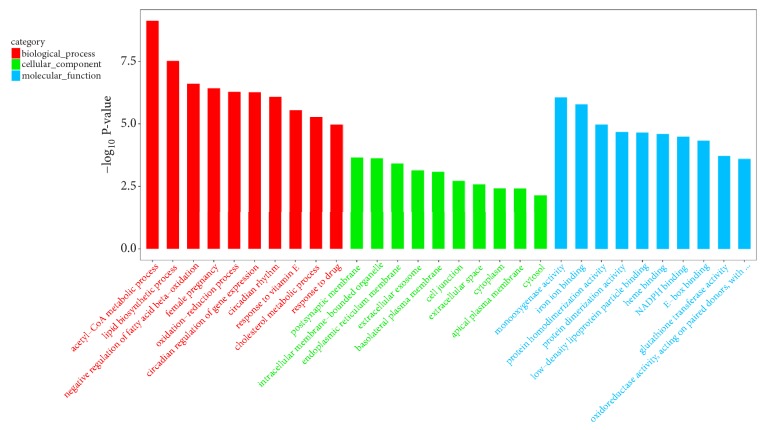
Compared with the normal control group, GO analysis result of YDCS model group.

**Figure 6:**
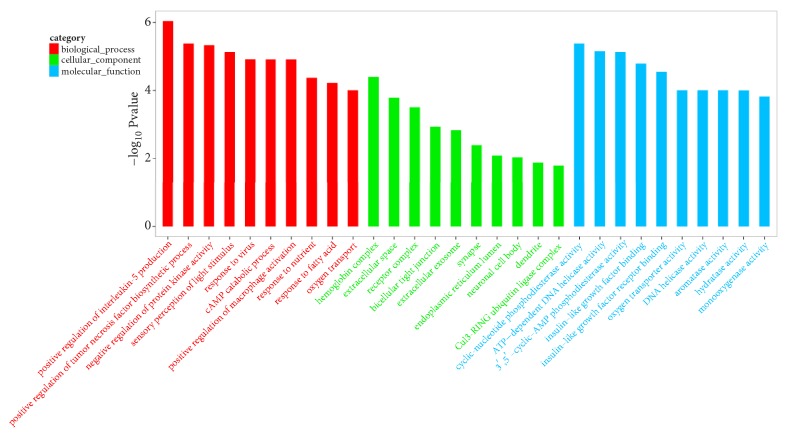
Compared with the YDCS model group, GO analysis result of RAWA treatment group.

In order to investigate the mechanism of the therapeutic effect of RAWA on YDCS, we used the second-generation sequencing to detect the whole-genomic level transcriptome of the rat liver DNA. The goal is to study the changes of gene expression level in metabolic-related genes. We chose Illumina HiSeq X Ten [31, 32] platform for our sequencing experiment. The results showed that the expression of metabolism-related genes in rats in YDCS model group presented a “reverse regulation” tendency. On the other hand, treatment with RAWA could reverse the trend of reduction in the expression of most metabolism-related genes (Figure 4(a)). More specifically, 38 typical metabolism-related genes were significantly downregulated in YDCS model group. After treatment with RAWA, 49 metabolism-related genes were upregulated (Figure 4(b)). GO analysis was performed to analyze the biological processes that influence the major differences in their genes, such as RNA metabolism, tissue development, and regulation of GTPase activity (Figure 5). Interestingly, RAWA downregulated genes associated with cell death; apoptotic, ERK1, and ERK2 cascades were downregulated. Meanwhile, RAWA can upregulate ATP binding, DNA repair, glucose metabolism, and cholesterol and lipid metabolism, which is contrary to the trend of changes in the YDCS model group (Figure 6). The GO functional annotations are mainly focused on several categories including glucose metabolism, lipid metabolism, inflammation, and amino acid metabolism. We found that after RAWA treatment, the expression of most metabolic genes increased, such as genes related to coenzyme metabolism, glucose metabolism, lipid metabolism and GTPase activity, and DNA replication initiation. On the contrary, the expression of these energy metabolism processes was downregulated in the YDCS model group. KEGG enrichment focusing on glucose, lipid, and amino acid metabolism showed that YDCS group and RAWA group were both enriched in PPAR signaling pathway.

Therefore, these data indicate that the use of RAWA to treat YDCS may play a role in regulating energy metabolism by downregulating apoptosis and upregulating glucose, lipid, and amino acid metabolism-related genes.

### 3.7. KEGG Pathway Enrichment Analysis of DEGs

**Figure 7:**
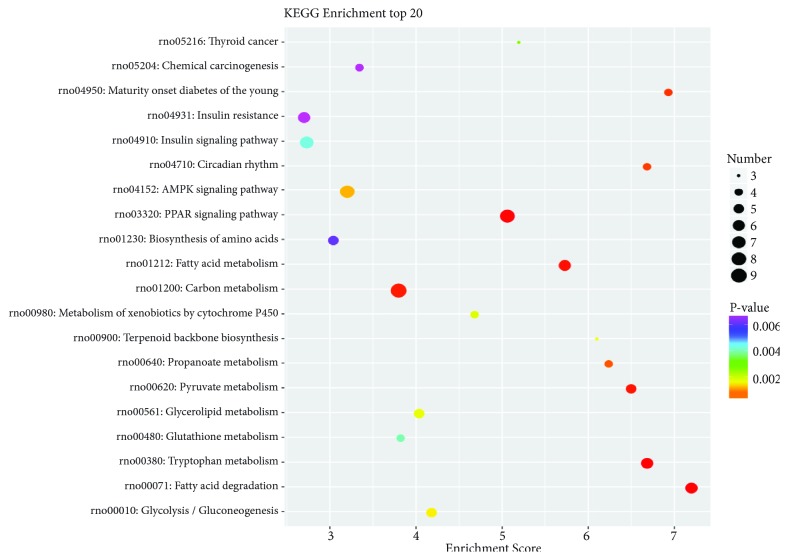
Compared with the normal control group, the rat metabolism was related to the YDCS in KEGG pathway.

**Figure 8:**
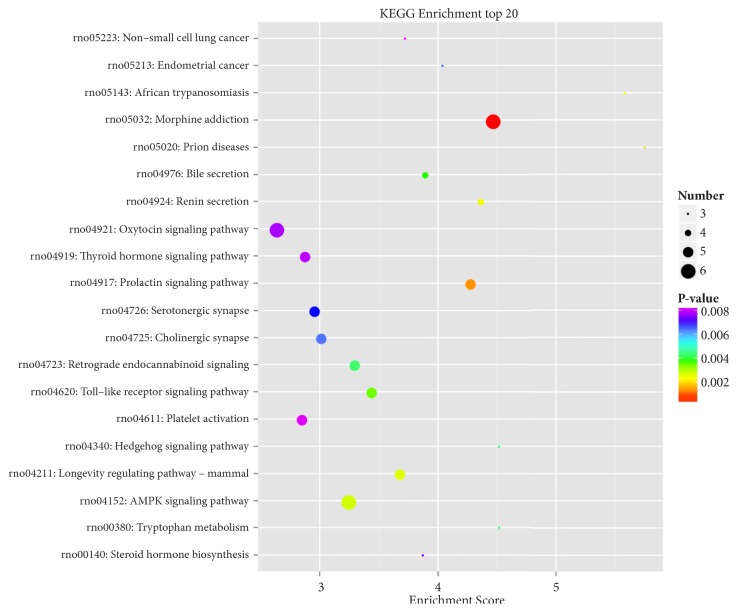
Compared with YDCS group, the rat metabolism was related to the RAWA in KEGG pathway. KEGG concentration analysis top 20 (the pathway entry with the corresponding number of corresponding genes greater than 2, according to the corresponding -log_10_ P-value of each entry). KEGG is the pathway of the main public database; using KEGG pathway analysis was carried out on the differences in gene database (in combination with the results KEGG comments), using hypergeometric inspection method to calculate gene enrichment of significant difference in each entry pathway. The result of the calculation will return a significant P-value with a small P-value indicating that the difference gene is enriched in the pathway. The X-axis in the figure represents the enrichment score, and the larger the bubbles, the more different the genes included in the entry. The color of the bubbles is changed by red-blue-green and yellow-yellow, which is rich in P-value.

Figures 7 and 8 showed significantly enriched pathways of DEGs through KEGG enriched analysis. DEGs of the model group were enriched in fatty acid degradation, PPAR signaling pathway, and AMPK signaling pathway (Table 1). In the RAWA treatment group, Wnt, cAMP, AMPK, and PPAR signaling pathway were enriched (Table 2), all related to energy metabolism.

Others, such as Toll-like receptor, p53 and Jak-STAT signaling pathway, Bile secretion, and calcium signaling pathway, were related to immunity.

Taken together, this KEGG pathway enrichment indicates that the pathology of YDCS and the effect of RAWA both focus on energy metabolism and immune responses.

### 3.8. Expression of Metabolism-Related Genes in YDCS Group and RAWA Treatment Group

Compared with normal model group, 96 genes showed most significant differences in expression levels among genes related to metabolism. Compared with YDCS model group, 59 genes had most significant differences among genes related to metabolism (Figure 4), 28 of which were biological processes, such as fatty acid metabolism, nutrient and glucose transport and catabolism, cAMP decomposition, protein kinase activity, regulation of glycoproteins, regulation of lipid and triglyceride biosynthesis. 31 of the 59 genes are responsible for molecular functions, including lipid metabolism, regulatory responses to GTP, AMP, and ADP, cyclic nucleotide phosphodiesterase activity, ATP-dependent DNA helicase activity, monooxygenase and hydrolase activity (Figure 7).

### 3.9. GO Analysis of Significant Differential Energy Metabolism-Related Genes in YDCS Group and AWA Treatment Group

According to the GO classification criteria, the YDCS group possessed energy metabolism gene functions compared with normal control group. The AWA treatment group possessed energy metabolism (concentration) gene functions compared with YDCS model group. Among those genes, 20 genes were related to glucose metabolism, 26 genes were related to lipid metabolism, and 35 genes were related to amino acid metabolism (Tables 3 and 4).

### 3.10. Results of Quantitative Real-Time PCR

**Figure 9:**
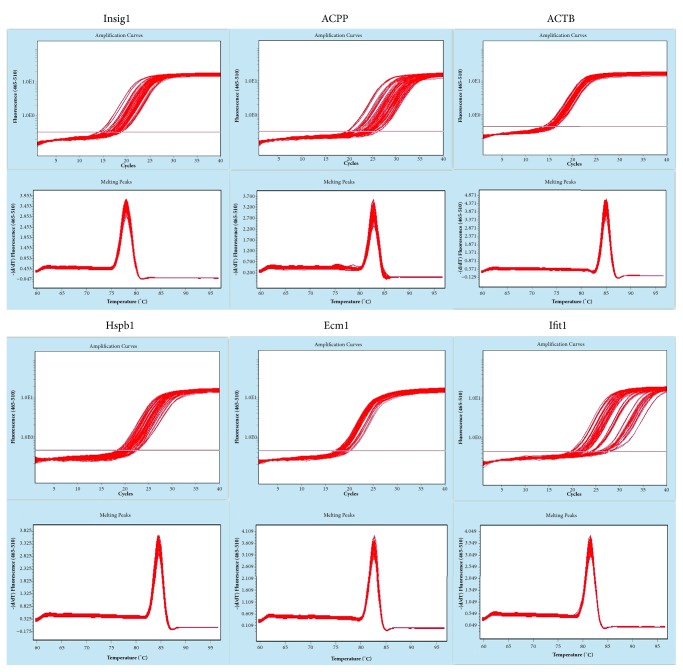


To confirm RNA-seq results, qRT-PCR was performed on Hspb1, Ecm1, Ifit1, Insig1, and Acpp being 0.1884, 0.8315, 2.3439, 5.0865, 0.1174, respectively, in the model group. And in RAWA group, the 2^−ΔΔCt^ values were 6.4745, 2.2208, 0.0521, 0.3910, and 0.3234, respectively (Table 5). The values, being consistent with the study of transcriptome sequencing technique, indicated the reliability of the results (Figure 9).

## 4. Discussion

The common feature of YDCS is the change of energy metabolism and weakened immunity, resulting in significant loss of body weight, drop in food/water intake and temperature, and reduced cold resistance. The pathogenic causes of YDCS are in accordance with the record of the ancient Chinese medicine classic* Nei jing*, which said that too much cold herb intake is harmful to Yang. The basic process of YDCS development is as follows: take too much cold herb, hurt the body righteousness, and result in damage of lung, spleen, kidney, liver, and other organs, poor circulation of blood, fatigue, chills, laziness, long-time lying, lack of movement, reduced water intake, loose stools, and other symptoms [2]. It can be divided into two major categories: Yang-deficiency (YD) constitution and the YDCS stage due to the development of the disease. In YD constitution, due to congenital deficiency, deficiency of kidney Yang, impaired function of lung, spleen, liver, and kidney, and other causes of deficiency of Yang, the body develops glucose-6-phosphate dehydrogenase [5], G protein (by controlling ATP, ADP, AMP, and GTP), and T cell immune functions, etc. reduction. According to this theory, in this experiment, we used a traditional cold compound formula of Chinese medicine to replicate the model of YDCS under the guidance of the comprehensive diagnosis and treatment of “Nature-Humanity Unity” proposed by Nei jing. Based on the difference in infrared thermal sensitivity of various parts of the human body, we utilized infrared imaging to detect the temperature changes in the YDCS model group and the RAWA treatment group. The principle of detection agrees with the traditional Chinese medicine diagnosis principle “inference of the inside comes from the outside”. As a result, we found that the YDCS model showed a reduction in energy metabolism represented by a drop of temperature, cold-tolerance, and basal metabolism on a macroscopic scale. After treatment with RAWA, the reduced energy metabolism increased significantly. Because these changes are closely related to the change of metabolic function in the human body, the transcriptome sequencing was used to sequence the whole genome of the liver, which is the main organ of metabolism. The sequencing results became more specific after the “noise” is excluded.

The emergence of YDCS because of the body's energy metabolism dysregulation was represented by glucose metabolism, lipid metabolism, and amino acid metabolism. Reduction by different degrees occurred in these metabolic functions causing further pathological changes in the body's endocrine and immunity. YDCS is a chronic pathological process, and it is closely related to PPAR signaling pathway and downregulation of the target genes CPT-1, FABP4 (Supplementary Figure 3) [33]. Therefore, as a typical mild Chinese medicine with few side effects, RAWA is suitable for long-term use for prevention and treatment of YDCS by focusing on various pathogenic and influencing factors in the early stage.

RA belongs to a kind of classical Chinese medicine with mild properties and warming effects [29]. Modern pharmacology shows that RA contains RA polysaccharides, astragaloside, flavonoids, alkaloids, vitamins, and other active ingredients [34]. RA membranaceus is widely used in clinical treatment of digestive system, respiratory system, cardiovascular diseases, endocrine diseases, and metabolic diseases. It has an enhancing effect on regulating energy metabolism and immune and metabolic functions [35]. RA also showed advantages including significant treatment effect, no toxic side effects, and less adverse reactions [36–40]. Previous tests showed that making RA into water abstract form can better unleash its function. This study found that RA can significantly increase the body weight, temperature, basal metabolism, and cold resistance of YDCS model rats. Through pharmacodynamic studies, it has been found that RA has a beneficial effect on the generation, storage, and use of energy in the body. Combined with the transcriptome results, the effect of RA membranaceus on energy metabolism may be due to the action on PPAR signaling pathway, resulting in the upregulation of the target gene CPT-1 and FABP4 (Supplementary Figure 4). RA can also enhance production of heat and promote the reduced energy metabolism in the YDCS model rats. These benefits are the specific manifestation of its warming effect [32, 33].

In the transcriptome sequencing, the major differential genes and pathway enrichment have shown that energy metabolism represented by carbohydrates, lipids, and amino acid metabolism is the core of the analysis of YDCS pathogenesis and RAWA treatment mechanism. RAWA upregulates lipid metabolism represented by ap2 (Fabp4) under the action of cytochrome P450 family member Cyp1a1 in vivo. Activation of PPAR signaling pathway (path: rno03320) and MAPK signaling pathway (path: rno04010) through activation of FABP resulted in the activation of PPAR*α* and PPAR*γ* pathways and significant downregulation of Scd1. This leads to increased lipid metabolism and upregulation of CPT-1 and ap2 (FABP4), which further affect AMPK signaling pathway (path: rno04152) in lipolysis. Also, the oxidation of fatty acids enhances lipid metabolism. All these effects will further influence the enhancement of the immune and endocrine system.

Among the molecules mentioned above, SCD-1 (stearoyl-CoA desaturase) is a key enzyme in lipid metabolism. It can catalyze the formation of monounsaturated fatty acids from saturated fatty acids, activate adenylate-activated protein kinases, and affect the synthesis of cholesterol and triglycerides, increasing fatty acid oxidation mostly in the liver, heart, and skeletal muscles [41]. It can affect the heat production of skeletal muscles as well [42, 43]. Furthermore, inflammation and cell stress response are regulated by the activation of MAPK signaling pathway and NF-*κ*B signaling pathway [44, 45]. The decrease of SCD-1 after the treatment with RA water decoction can reduce fat accumulation, increase heat production and lipid metabolism [46], accelerate fatty acid decomposition, and promote energy metabolism of the body.

CPT1 is a key enzyme in the fatty acid oxidation process. It is located in the mitochondrial outer membrane and is the rate-limiting enzyme in the fatty acid *β* oxidation process [47]. CPT1 can be divided into three subtypes, increase FFA (nonesterified fatty acid), promote lipolysis.

Among which CPT1A has the strongest activity in the liver [48]. Therefore, CPT1A can regulate the oxidation of fatty acids in the body to a greater extent and increase the rate of fatty acid oxidation, which plays an important role in the changes of energy metabolism in the body.

Ap2 (Fabp4) belongs to the FABP family, which are a family of fatty acid-binding proteins that bind to hydrophobic lipids and carry out intracellular transportation. It binds reversibly to saturated and unsaturated long-chain fatty acids. Fabp4 executes its function in regulating the metabolism and transport of fatty acids through the promoter-binding elements PPRE1 and PPRE2 presented in PPAR*γ* pathway, leading to the important role in the body's energy metabolism [47, 48]. More literature has shown that Fabp4 has an essential effect on the metabolic syndrome. The increased Fabp4 expression can induce the upregulation of acetyl-CoA cholesterol acyltransferase 1 (ACAT1) gene expression [49], but it can also regulate the gene expression of ATP binding cassette transporter A1 (ABCA1) and hormone-sensitive lipolytic enzyme (HSL) in the opposite direction, resulting in decreased degradation of triglycerides and cholesterol. It is worth noting that literature studies have pointed out that overexpression of FABP4 can cause increased metabolism and inflammatory enhancement. The latter can lead to atherosclerosis and other related diseases. However, our experiment is comparing with the metabolically deficient model rats of YDCS. The focus is on the recovery to normal level, so it does not involve the overexpression of FABP4.

Based on symptoms and the macroscopic/microscopic observations of temperature and metabolism, combined with modern research, we discussed the effects of RA and YDCS on energy metabolism. From the genetic level, changes in key pathway PPARs are the main entry point. PPAR signaling pathway is the key metabolic pathway and CPT-1, FABP4 are the key target genes that regulate lipid metabolism. CPT-1, FABP4 are also key targets for the treatment of metabolic and immune diseases. According to transcriptome sequencing results, in the YDCS model group, PPAR pathway was activated, in which CPT-1 and FABP4 were significantly reduced, while after RAWA treatment, CPT-1 and FABP4 in the PPAR pathway were significantly upregulated, indicating that RAWA acts on these targets. RAWA showed a clear adjusting role in metabolic system. This role may take effect through the role of PPAR signaling pathway and the target genes CPT-1 and FABP4 by improving metabolism reduced by YDCS. In addition, FABP4 upregulation can enhance basal metabolism and improve the metabolic dysfunction caused by reduced metabolism in YDCS. The purpose of enhancing immunity can be achieved by enhancing the expression of inflammatory factors such as TNF-*α*, IL-1, and IL-6. All the effects mentioned above will achieve the ultimate purpose of treatment of cold syndrome [50].

## 5. Conclusion

In summary, the mechanism of YDCS pathology is energy metabolism deficiency caused by PPAR signaling pathway and target genes CPT-1, FABP4. RAWA had well effects that could upregulate CPT-1, FABP4 via multiple pathways to promote energy metabolism of rats with the YDCS syndrome, especially through the PPAR signaling pathway. RAWA can also further upregulate immunity. These effects indicate the tonic and warm properties of RAWA. Further study is needed for the details of the regulative mechanism.

## Figures and Tables

**Table 1:** KEGG pathway enrichment analysis DEGs of the model group related to energy metabolism.

Id	Term	P-val	Enrichment score	Gene Symbol
path:rno00071	Fatty acid degradation	1.53E-05	7.194942	LOC100911186;Acat2;Eci1;Cpt1b;
				Cyp4a1;Aldh1b1
path:rno03320	PPAR signaling pathway	2.47E-05	5.055905	Plin5;Cyp8b1;Gk;Fabp5;Cpt1b;Scd1;
				Cyp4a1;Dbi
path:rno00620	Pyruvate metabolism	9.89E-05	6.495434	Acat2;Pklr;Acss2;Aldh1b1;Acacb
path:rno01212	Fatty acid metabolism	7.17E-05	5.726587	LOC100911186;Acat2;Fasn;Fads1;
				Cpt1b;Scd1
path:rno00061	Fatty acid biosynthesis	0.00234	7.194942	Scd1;Acacb
path:rno00380	Tryptophan metabolism	2.55E-05	6.681018	Kynu;LOC100911186;Acat2;Cyp1a1;
				Ddc;Aldh1b1
path:rno04152	AMPK signaling pathway	0.000848	3.197752	Srebf1;Creb3l1;Fasn;Ccnd1;Cpt1b;
				Scd1;Hmgcr;Acacb
path:rno00010	Glycolysis / Gluconeogenesis	0.001161	4.175636	Gck;Pklr;Acss2;Aldoc;Aldh1b1
path:rno00561	Glycerolipid metabolism	0.001397	4.031649	Gpat3;Gk;Tkfc;Pnpla3;Aldh1b1
path:rno00980	Metabolism of xenobiotics by cytochrome P450	0.001503	4.676712	LOC102550391;Cyp1a1;Gstt1;Gsta3
path:rno00480	Glutathione metabolism	0.003734	3.817724	LOC102550391;Gpx2;Gstt1;Gsta3
path:rno01230	Biosynthesis of amino acids	0.005878	3.036826	Tkt;Pklr;Pycr1;Aldoc;Gpt
path:rno04922	Glucagon signaling pathway	0.007072	2.922945	Gck;Slc2a2;Creb3l1;Cpt1b;Acacb
path:rno00100	Steroid biosynthesis	0.007219	4.922855	Fdft1;Tm7sf2
path:rno00220	Arginine biosynthesis	0.007219	4.922855	Gpt;Nos1
path:rno00340	Histidine metabolism	0.010949	4.251557	Ddc;Aldh1b1
path:rno01040	Biosynthesis of unsaturated fatty acids	0.01395	3.89726	Fads1;Scd1
path:rno00330	Arginine and proline metabolism	0.016333	3.05003	Pycr1;Aldh1b1;Nos1
path:rno04146	Peroxisome	0.027864	2.338356	Crot;Ephx2;Ech1;Pex11a

**Table 2:** KEGG pathway enrichment analysis DEGs of the AWA treatment group related to energy metabolism and immunity.

Id	Term	P-val	Enrichment score	Gene Symbol
path:rno04152	AMPK signaling pathway	0.002426	3.242165	Foxo3;Ccnd1;Cpt1b;Scd1;Adipor2;Pik3r1
path:rno00380	Tryptophan metabolism	0.004174	4.515873	Inmt; Cyp1a1; Ddc
path:rno00140	Steroid hormone biosynthesis	0.007262	3.870748	RGD1559459;Cyp1a1; Cyp2d4
path:rno04310	Wnt signaling pathway	0.021086	2.30738	Prickle2;Prkacb; Ccnd1; Nfatc4; Lrp6
path:rno04024	cAMP signaling pathway	0.024854	2.084249	Adora2a; Prkacb; Pde4d; Adcyap1r1; Pde4c; Pik3r1
path:rno00980	Metabolism of xenobiotics by cytochrome P450	0.02489	3.161111	RGD1559459; Cyp1a1
path:rno00982	Drug metabolism - cytochrome P450	0.026553	3.084011	RGD1559459;Fmo2
path:rno03320	PPAR signaling pathway	0.029248	2.563063	Fabp4; Cpt1b; Scd1
path:rno04922	Glucagon signaling pathway	0.037441	2.370833	Prkacb; Gnaq; Cpt1b
path:rno01212	Fatty acid metabolism	0.041939	2.580499	Cpt1b;Scd1
path:rno00340	Histidine metabolism	0.046618	2.873737	Ddc
path:rno00360	Phenylalanine metabolism	0.035579	3.327485	Ddc
path:rno04620	Toll-like receptor signaling pathway	0.00323	3.43599	Mapk12; Cxcl10; Lbp; Pik3r1
path:rno04115	p53 signaling pathway	0.017262	3.010582	Sesn3; Igfbp3; Ccnd1
path:rno04750	Inflammatory mediator regulation of TRP channels	0.019943	2.554433	Prkacb; Mapk12; Gnaq; Pik3r1
path:rno04630	Jak-STAT signaling pathway	0.023181	2.257937	Cish; Ccnd1; Il13ra1; Stam2; Pik3r1
path:rno04670	Leukocyte transendothelial migration	0.030841	2.278278	Cd99; Mapk12;Cldn15; Pik3r1
path:rno04068	FoxO signaling pathway	0.041079	2.107407	Mapk12; Foxo3; Ccnd1; Pik3r1

**Table 3:** GO analysis of significant differential energy metabolism related genes in YDCS group and RAWA treatment group.

The serial number	Gene name	GOid	Fold change	P-val	GO function
ENSRNOG00000003259	C1qtnf1	GO:0051897	0.414337	0.000326	Sugar and protein metabolism
ENSRNOG00000010438	Cpt1b	GO:0006635	0.435098	0.016545	Lipid metabolism
ENSRNOG00000014532	Lbp	GO:0001889	0.46867	0.000321	Lipid metabolism
ENSRNOG00000003028	Dnah17	GO:0005524	0.417407	0.02197	Energy metabolism
ENSRNOG00000020369	Igf2	GO:0045953	0.34108	0.035391	Sugar acid metabolism
ENSRNOG00000030154	Cyp4a2	GO:0005504	0.084145	0.035647	Lipid metabolism
ENSRNOG00000010438	Cpt1b	GO:0015909	0.435098	0.002426	Lipid metabolism
ENSRNOG00000031233	Mapk12	GO:000552	0.443239	40.009524	Amino acid metabolism
ENSRNOG00000014524	S1pr3	GO:0005515	0.392174	0.009524	Amino acid metabolism
ENSRNOG00000001302	Adora2a	GO:003081	0.482049	0.013993	Sugar metabolism
ENSRNOG00000020585	Tbxa2r	GO:000718	0.46712	0.013993	Amino acid metabolism
ENSRNOG00000003033	Plcd3	GO:000487	0.401252	0.013993	Lipid metabolism
ENSRNOG00000036828	Pde1b	GO:0043025	0.123816	0.013993	Amino acid metabolism
ENSRNOG00000012098	Adcyap1r1	GO:0030819	0.462201	0.041939	Amino acid metabolism
ENSRNOG00000019518	Pde4c	GO:0006198	0.305552	0.041939	Amino acid metabolism
ENSRNOG00000010805	Ap2	GO:0008289	3.398621	0.040759	Lipid metabolism
ENSRNOG00000010438	CPT-1	GO:0015909	2.298333	0.016545	Lipid metabolism
ENSRNOG00000023546	Hspb1	GO:2001028	8.084827	0.00208	Heat shock protein
ENSRNOG00000014882	Fgf11	GO:0008083	2.089544	0.025097	growth factor activity

**Table 4:** The genes of cytochrome P450 family were significantly downregulated, suggesting that the YDCS model can reduce lipid metabolism and biotransformation function in rats. On the contrary, RAWA can increase lipid metabolism and biotransformation function.

The serial number	Gene name	Fold change	P-val	GOid	GO function
ENSRNOG00000055078	Cyp4b1	0.42533	0.03924	GO:0055114	Energy metabolism
ENSRNOG00000019500	Cyp1a1	0.253766	0.0269	GO:0032496	Lipid metabolism
ENSRNOG00000002134	Cyp4a2	2.497622	0.00689	GO:0003924	Amino acid metabolism
ENSRNOG00000032261	Cyp2d4	0.463307	0.00951	GO:0019369	Lipid metabolism
ENSRNOG00000003510	Fmo2	0.285916	0.02557	GO:0006739	NADP metabolism
ENSRNOG00000029478	Cyp4f39	2.243402	0.0016	GO:0016705	Energy metabolism
ENSRNOG00000042714	RGD1559459	2.439187	0.03076	GO:0052696	NADP metabolism

**Table 5:** The primers of selected genes for qRT-PCR

GenBank accession number	Gene symbol	Forward primer (5 -3 )	Reverse primer (5 -3 )
ENSRNOG00000023546	Hspb1	GGAGATCACCATTCCGGTC	GCAAGCTGAAGGCTTCTAC
ENSRNOG00000021166	Ecm1	TCTTGACCGTTGACCTTAGC	GCCAGGGATCTGTTTATGC
ENSRNOG00000019050	Ifit1	CCTTTGCCTGGAGGAAACTA	CACAAGCCGGACATTCTG
ENSRNOG00000006859	Insig1	GTCTCATCAGTGTGGGAACTA	CTGGGCTGTCAGTAAGATTG
ENSRNOG00000011820	Acpp	CAAGCCAAGGAGTTGAAGT	GGCCACGAGGATTCCTTA
NM_031144	ACTB	CCACCATGTACCCAGGCATT	CGGACTCATCGTACTCCTGC

## Data Availability

The data used to support the findings of this study are available from the corresponding author upon request.

## Supplementary Materials

Supplementary Materials Supplementary Figure 1:* Morphological observation of rats*: Compared with the normal control group, rats in YDCS model group appeared to have an addiction of lying, messy and dry hair, squinting, listless, pale and partial darkness on lips and paws, clear urine in large amount, loose stools, etc. Supplementary Figure 2:* Morphological observation of rats*: After 7 days of the treatment with RA water abstract, the overall condition of the animals improved significantly. Hair became smoother and shiner; rat activity increased. Lips, stools forming became moderate; nails turned white and pink; urine became of normal color. Supplementary Figure 3:* The change of PPAR signal pathway in YDCS model*: The PPAR signal pathway is an important pathway for metabolism and immunity. In YDCS pathological process, rat glucose metabolism, lipid metabolism, and amino acid metabolism reduction were disordered. The body's energy metabolism dysregulation of rats reflects the abnormal PPAR signal pathway. It is closely related to PPAR signaling pathway and downregulation of the target genes CPT-1, FABP4. Supplementary Figure 4:* The change of PPAR signaling pathway in RAWA treatment group*: Combined with the transcriptome results, the effect of RA membranaceus on energy metabolism may be due to the action on PPAR signaling pathway, resulting in the upregulation of the target gene CPT-1 and FABP4. RA can also enhance production of heat and promote the reduced energy metabolism in the YDCS model rats. These benefits are the specific manifestation of its warming effect. RAWA upregulates lipid metabolism represented by ap2 (Fabp4) under the action of cytochrome P450 family member Cyp1a1 in vivo. Activation of PPAR signaling pathway (path: rno03320).
