# Supplementary material for: The Effects of Radix Astragali Water Abstract on Energy Metabolism in Rat Yang-Deficiency Cold Syndrome Model through PPAR Signaling Pathway
Source: Evid Based Complement Alternat Med. 2018 Nov 1;2018:9194362. doi: 10.1155/2018/9194362 (PMC6241352; doi:10.1155/2018/9194362)

The attachment

1. Supplementary fig. 1

Morphological observation of rats

Compared with the normal control group, rats in YDCS model group appeared to have an addiction of lying, messy and dry hair, squinting, listless, pale and partial darkness on lips and paws, clear urine in large amount, loose stools, etc.

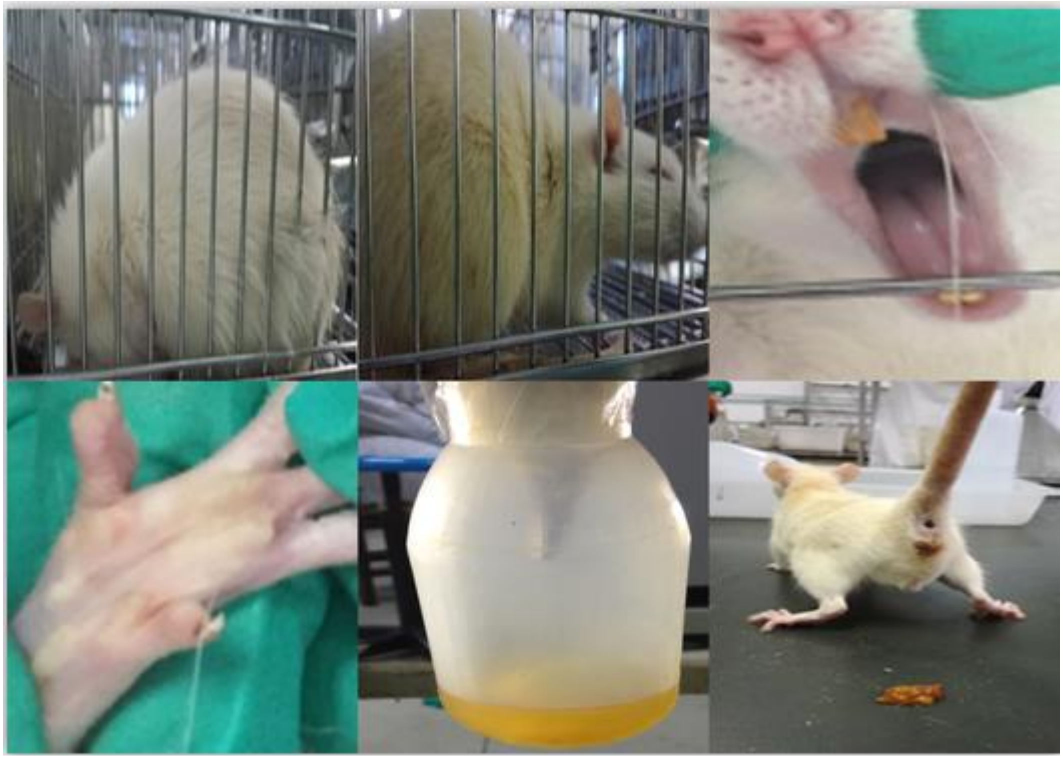

2. Supplementary fig. 2

Morphological observation of rats

After 7 days of treatment with RA water abstract, the overall condition of the animals improved significantly. The total activity, hair became smoother and shiner, good mental health and increased activity, Lips, stools forming and became moderate, nails turned white and pink., improvement in urine color, etc.

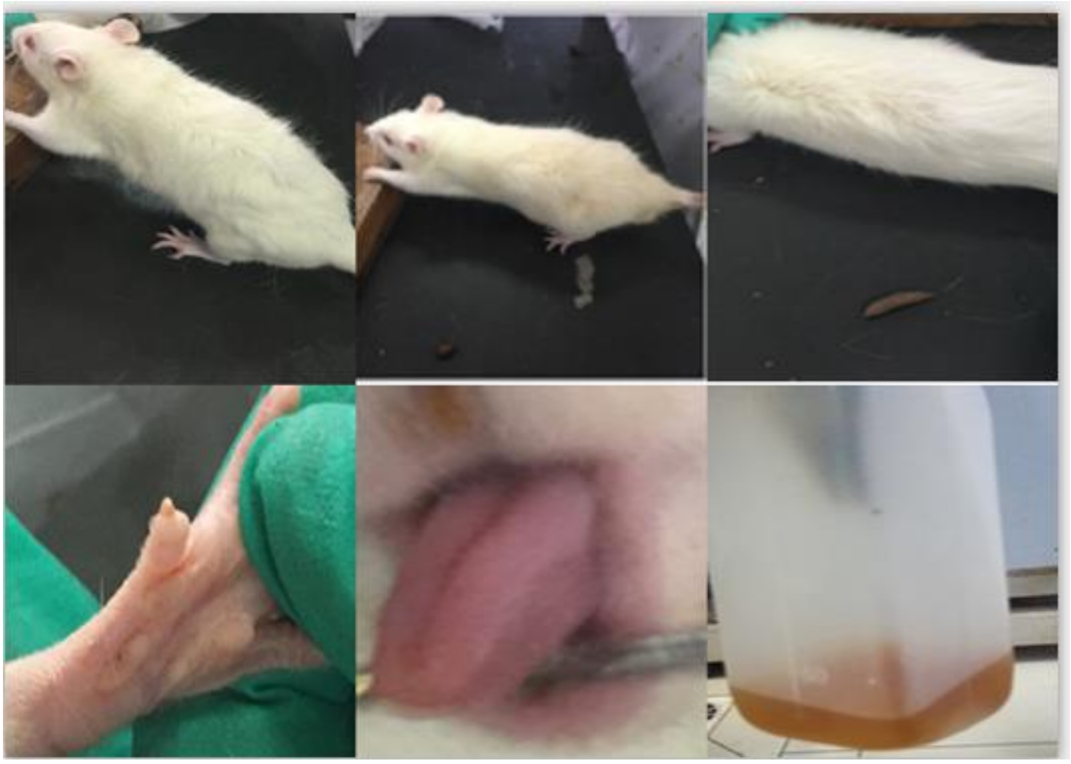

3. **Supplementary fig. 3**

The change of PPAR signal pathway in YDCS model

The PPAR signal pathway is an important pathway for metabolism and immunity, in YDCS pathological process, body's energy metabolism dysregulation represented by glucose metabolism, lipid metabolism, and amino acid metabolism reduction, reflect in PPAR signal pathway. It is closely related to with PPAR signaling pathway and downregulation of the target genes CPT-1, FABP4.

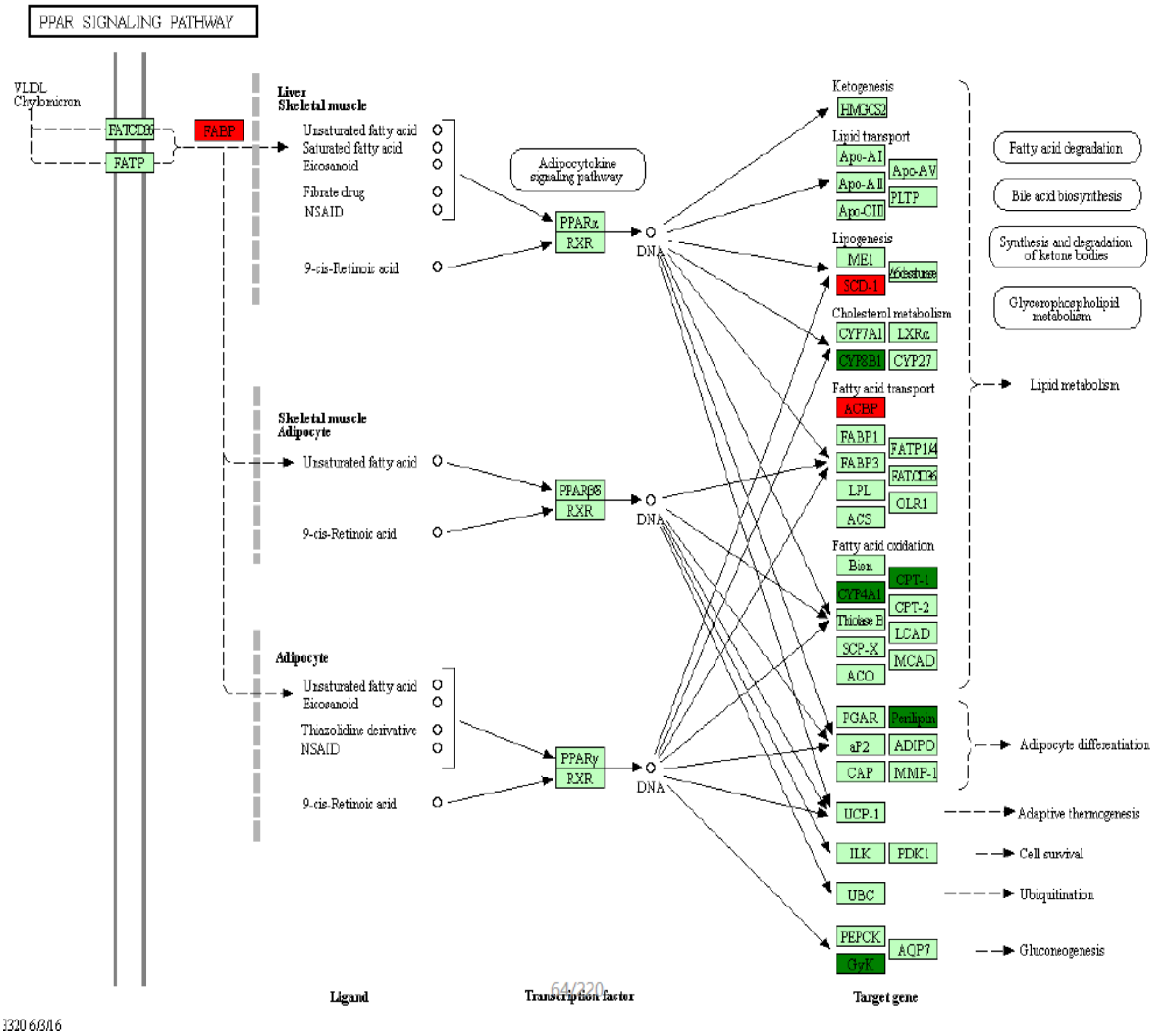

4. Supplementary fig. 4

The change of PPAR Signaling Pathway in RAWA treatment group

Combining with the transcriptome results, the effect of RA membranaceus on energy metabolism may be due to the action on PPAR signaling pathway, resulting in the upregulation of the target gene CPT-1 and FABP4. RA can also enhance production of heat and promote the reduced energy metabolism in the YDCS model rats. These benefits are the specific manifestation of its warming effect. RAWA upregulates lipid metabolism represented by ap2 (Fabp4) under the action of cytochrome P450 family member Cyp1a1 in vivo. Activation of

PPAR signaling pathway (path: rno03320).

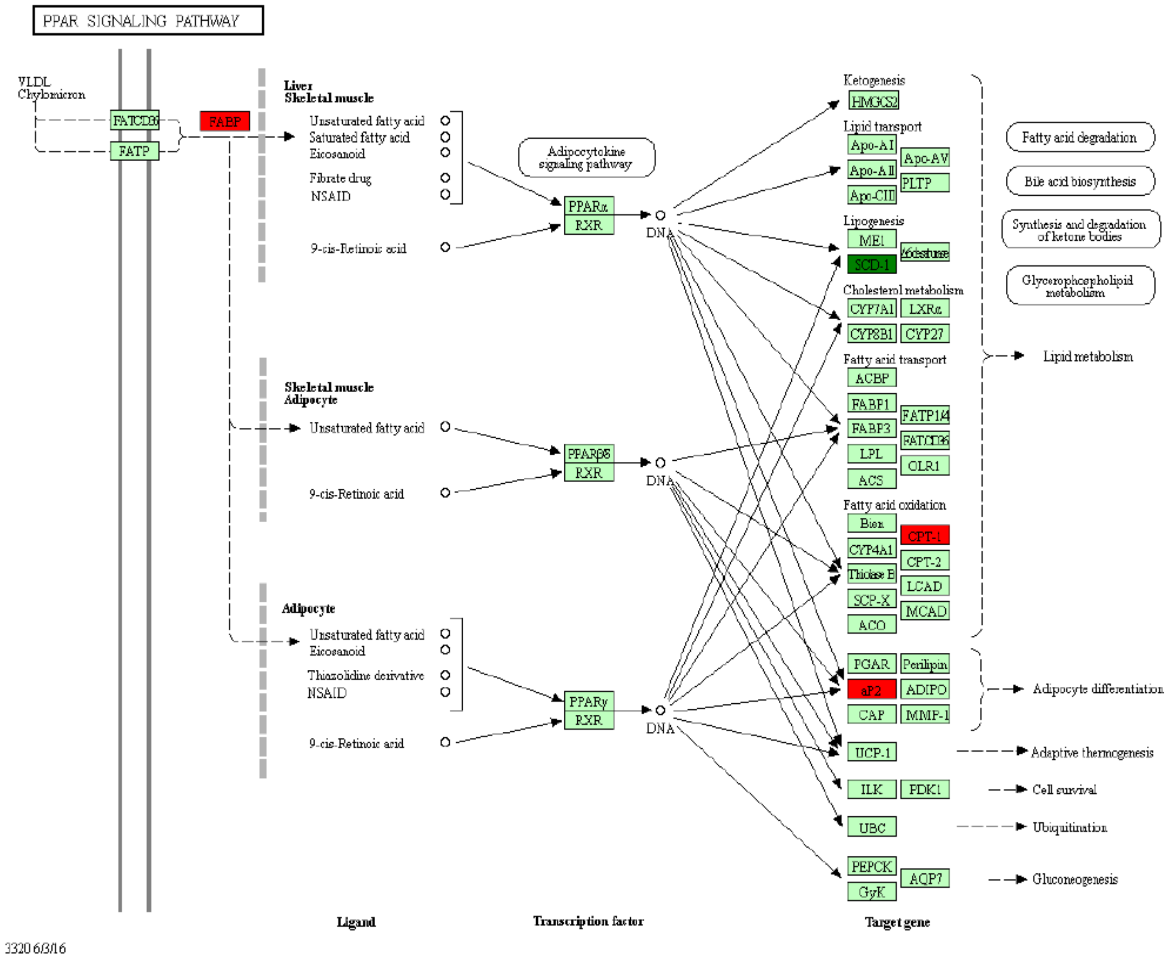

Supplement: Supplementary Materials — Supplementary Figure 1: Morphological observation of rats: Compared with the normal control group, rats in YDCS model group appeared to have an addiction of lying, messy and dry hair, squinting, listless, pale and partial darkness on lips and paws, clear urine in large amount, loose stools, etc. Supplementary Figure 2: Morphological observation of rats: After 7 days of the treatment with RA water abstract, the overall condition of the animals improved significantly. Hair became smoother and shiner; rat activity increased. Lips, stools forming became moderate; nails turned white and pink; urine became of normal color. Supplementary Figure 3: The change of PPAR signal pathway in YDCS model: The PPAR signal pathway is an important pathway for metabolism and immunity. In YDCS pathological process, rat glucose metabolism, lipid metabolism, and amino acid metabolism reduction were disordered. The body's energy metabolism dysregulation of rats reflects the abnormal PPAR signal pathway. It is closely related to PPAR signaling pathway and downregulation of the target genes CPT-1, FABP4. Supplementary Figure 4: The change of PPAR signaling pathway in RAWA treatment group: Combined with the transcriptome results, the effect of RA membranaceus on energy metabolism may be due to the action on PPAR signaling pathway, resulting in the upregulation of the target gene CPT-1 and FABP4. RA can also enhance production of heat and promote the reduced energy metabolism in the YDCS model rats. These benefits are the specific manifestation of its warming effect. RAWA upregulates lipid metabolism represented by ap2 (Fabp4) under the action of cytochrome P450 family member Cyp1a1 in vivo. Activation of PPAR signaling pathway (path: rno03320). [file 9194362.f1.pdf]
